# Patients' attitudes towards switching to national volume-based procurement (NVBP) Drugs—a qualitative investigation in Wuhan, China

**DOI:** 10.1186/s12913-023-09077-4

**Published:** 2023-01-21

**Authors:** Jinyi Tao, Shihong Li, Qiling Xu, Shiwei Gong, Fang Zeng

**Affiliations:** 1 Department of Pharmacy, Union Hospital, Tongji Medical College, Huazhong University of Science and Technology, Wuhan, China; 2 School of Pharmacy, TongJi Medical College, Huazhong University of Science and Technology, Wuhan, China

**Keywords:** National Volume-Based Procurement (NVBP), Patients, Attitude, Qualitative Investigation, Policy

## Abstract

**Background:**

The implementation of the NVBP policy has generated considerable reductions in drug procurement prices and an increase in the market share of the NVBP drugs.This study aimed to investigate patients' attitudes towards switching to drugs of national volume-based procurement (NVBP) and identify their underlying influencing factors in Wuhan, China.

**Methods:**

A total of 21 eligible patients from the Wuhan Union Hospital who were switched to NVBP drugs between January 2022 and May 2022 were included in our study. Semi-structured face-to-face interviews were conducted to collect interview information and the interview data was analyzed by the Colaizzi seven-step method.

**Results:**

Twenty-one semi-structured face-to-face interviews were conducted. The duration of each interview was 25–35 min and three themes related to patients' attitudes and their influencing factors were extracted, including (1) Patients' perception of the NVBP drugs; (2) Family and social influence to patients; (3) Medication habits of patients. This study found: 1) 71.4% patients (15/21) showed a positive attitude towards switching to NVBP medicines; 2)80.9% patients (17/21) have felt a significant reduction in their medication cost after the implementation of the NVBP policy; 3)Advices from healthcare professionals and health insurance reimbursement policies showed great impacts on patients' attitude towards switching to NVBP drugs; 4)Attitudes towards switching to NVBP drugs varied considerably among patients with different severities of disease.

**Conclusion:**

The implementation of the NVBP policy has significantly reduced the cost of healthcare for patients and has been supported by71.4% (15 of 21) patients. However, some issues have been identified in the implementation of the policy in this study. Health professionals in general need to contribute more efforts to improve patients' preconceptions about the NVBP drugs and boost their confidence in the NVBP drugs.

## Introduction

With the rapid development of medical technology in this aging population, the world is faced with the dilemma of burden of healthcare spending [1, 2]. The proportion of expenditure on medicines in health care expenditure continues to grow in recent decades [3–6]. Group purchasing organizations (GPOs) in the United States have played a major role in reducing healthcare costs and improving the efficiency of the healthcare supply chain for over 100 years, which saves approximately $36 billion annually for the entire healthcare system [7, 8]. The vast majority of medical institutions decision makers will expand the use of GPOs in order to control the growth of healthcare cost [9]. In recent years, the health expenditure in China has also increased rapidly with an overall upward trend of the proportion (percentage of GDP) of fiscal expenditure [10]. The cost on drugs in China was significantly higher accounting for 30–40% of the total healthcare costs, compared to 5%-20% in economically developed countries [11]. However, hospital outpatients' average per-visit medicine costs exceed 40% [12]. In order to limit the rapid growth of healthcare costs in China, the Chinese State Council approved a pilot program of NVBP (so-called “4 + 7” policy) in January 2019 [13], and the substitution of NVBP drugs in China has become an irresistible trend.

The policy of the NVBP drugs is to build a unified national drug procurement market under the unified organization of the National Healthcare Security Administration, relying on provincial centralized drug procurement platforms. The healthcare security department adopts a strategic purchasing approach to exchange quantity for price, which can improve the efficiency of procurement and ultimately obtain reasonable prices for drugs [14, 15]. In the policy, original branded drugs or generic drugs that have passed the consistency evaluation of quality and efficacy are eligible to win the tender to become NVBP drugs. As of January 2022, there are 502 drugs in the list of NVBP drugs, of which there are 479 generic drugs and 23 original branded drugs. The implementation of the NVBP policy has generated considerable reductions in drug procurement prices and an increase in the market share of the NVBP drugs, with accumulated expenditure savings of over CNY 260 billion yuan over three years. Researches on the NVBP policy have also indicated a measurable reduction in drug prices, alleviating the burden of drug costs on patients [16, 17].

However, after the enforcement of the NVBP policy, some disadvantages were shown among medical staffs, such as the change in daily prescribing behavior and the unsatisfactory effects of the NVBP drugs [18]. There is also widespread concern about the rise in adverse reactions to the NVBP drugs [19]. Although the perceptions of Chinese pharmacists about the NVBP drugs have been studied [20], some other researchers have mentioned patients' doubts about the quality of the NVBP drugs and their restricted access to primary medications [21]. However, patients' attitudes towards switching to NVBP drugs have not been studied. As the consumers of the drugs, the recognition of the NVBP policy and feeling of switching to NVBP drugs would directly affect the public's evaluation of the policy and its promotion effect, which deserve the attention of researchers and policy makers. Therefore, we performed this study among the patients in Wuhan Union Hospital to investigate patients' attitudes towards switching to NVBP drugs and identify its underlying influencing factors.

## Methods

### Design

A qualitative descriptive design was adopted. This study used a qualitative descriptive design with the intention of understanding the attitudes of those patients who switched to NVBP drugs [22]. The data collection framework included semi-structured interviews to elicit information and also to enable patients to articulate their understanding of the switch to NVBP drugs [23]. This study strictly followed the Consolidated Criteria for Reporting Qualitative Studies (COREQ) checklist [24].

### Sampling and recruitment

Participants were recruited using purposive sampling among patients attending the Union Hospital affiliated to Huazhong University of Science and Technology. The hospital is a teaching hospital with 6,000-beds located in the central part of China. Participants must meet all the following criteria: 1) Diagnosed with one or more chronic diseases and prescribed at least one of the NVBP drugs. 2) History of switching to NVBP drugs. 3) Ability to clearly communicate with researchers. 4) Patients was informed prior to the interviews. Any individuals who did not wish to participate in the interview or who did not communicate well were excluded. Sampling and recruitment of study participants continues until data saturation. 18 samples were used for data analysis and the remaining 3 were used for saturation testing.

### Ethical considerations

This study was conducted in accordance with the Declaration of Helsinki [25]. The study protocol was reviewed and approved by the Institutional Review Board of Union Hospital, Tongji Medical College, Huazhong University of Science and Technology (UHCT22442). Informed consent was obtained from all subjects.

### Data collection and analysis

Data was collected during January 2022 to May 2022 with semi-structured face-to-face interviews and field notes. All interviews were conducted separately by two pharmacists who are both experienced pharmacists and trained interviewers. The duration of each interview ranged from 25 to 35 min, with an average interview duration of 31 min. Each interview was recorded and transcribed verbatim with the consent of the participant.

The outline of the interviews was built based on published papers on patients' perceptions of medication and problems encountered by pharmacists in their practical work [26, 27]. The interview outline included the following five questions: 1)Can you describe your medical, including medication history and experience with medication replacement? 2)What do you know about the NVBP policy and what changes has it brought to you? 3)What were the reasons for switching to NVBP medicines and what were your experiences and feelings after the switch? 4)Is it convenient for you to buy again and what is your route to buy again after the change? 5)What are your comments and suggestions? The questions in the interview outline are designed as open-ended questions that can be flexibly adjusted to the situation and encourage participants to express their real thoughts.

All recordings were transcribed verbatim after the interview within 24 h, which were then thoroughly reviewed separately by two researchers for several times to ensure accuracy. The transcriptions were translated into English and back-translated into Chinese to ensure consistency. Study subjects were coded as P1–P21. Two investigators independently completed the data analysis, coding and thematic analysis. Group discussions were conducted to form a unified opinion [28]. The Colaizzi seven-step [29, 30] approach was applied for data analysis, including: (1) Transcribing all recorded data; (2) Extracting significant descriptions; (3) Creating initial meanings; (4) Refining themes; (5) Describing each theme in detail; (6) Identifying the fundamental structure; (7) Returning analyzed data back to participants. The raw data was analyzed by the analysis software (Nvovo V.12) to further code it into sub-themes and themes. Some representative statements of the participants are extracted in this article. The investigators collected socio-demographic information from the participants.

### Trustworthy

Throughout the study, three measures were introduced to ensure the reliability of the study. Firstly, investigators maintained close communication with experts during the study. Secondly, the researcher collated with the participants after the data transcription to ensure the accuracy and consistency of information transmission. Finally, the researchers followed strictly the study protocols and research methods of qualitative research.

## Result

A total of 21 participants completed the interviews, involving 12 males and 9 females. 57.1% participants (12/21) have been taking medication for more than 5 years. The median age of the participants was 58 years old. The socio-demographic characteristics of the respondents are shown in Table 1.

**Table 1:** Socio-demographic characteristics of study participants

Variables	Frequency (N)
Age (in years)	
18–30	1
31–40	3
41–50	3
50–60	6
≥ 61	8
Gender	
Female	9
Male	12
Duration of taking medication (in years)	
≤ 5	9
6–10	3
> 10	9
Personal annual income (CNY: yuan)	
≤ 50000	4
50001–100000	7
100001–200000	7
> 200000	3
Medical insurance reimbursement	
Yes	17
No	4
Education level	
Junior High School and below	5
High School	5
Bachelor and above	11

After analyzing the content of the interviews, three themes related to patients' attitudes towards switching to NVBP drugs and the influencing factors were extracted: (1) Patients' perception of NVBP drugs; (2) Family and social factors of patients; (3) Medication habits of patients.

### Theme 1: Patients' perception of NVBP drugs

All participants (*n* = 21) mentioned their perceptions of the NVBP drugs, with the efficacy and safety of the medicines being of greatest concern.

#### Subtheme 1.1-Efficacy

There were five participants claimed that the efficacy of the NVBP drugs was equivalent to those brand drugs. The corresponding monitoring indicators were also the same affirming the efficacy of the NVBP drugs.

“I've been really well taking this medicine and my blood pressure is under control.” (P19)

However, two participants doubted the efficacy of the NVBP drugs because of fluctuations in the corresponding monitoring indicators after changing to the NVBP drugs.

“Since the doctor changed to use a domestic drug, my postprandial blood sugar get hard to controll, comparing with the brand ones.” (P6).

“After switching to a domestic drug, the glycemic controll was worse, and thus the acarbose was prescribed by the doctor” (P8).

Fourteen participants were worried about the efficacy of the NVBP drugs because it is difficult to monitor their own treatment indicators.

“If I have to switch to a domestic product, I will definitely have to judge whether the domestic product is as effective as the imported one, and this concern will bother me.” (P9).

“After taking domestic medication, I only came to check my blood lipids at the end of April and I was afraid that it would not be effective.” (P10).

#### Subtheme 1.2- Safety

Two participants expressed confidence in the NVBP drugs and did not feel any discomfort after taking the NVBP drugs for a period of time.

“I did not feel any physical discomfort after switching to the NVBP drug. I think the domestic quality of common drugs is already as good as the original drugs”(P8).

Adverse effects were a main concern for respondents(four participants), who felt that the "potential" adverse effects of NVBP drugs could cause some invisible side effects to the body compared to branded drugs.

“It is possible that the side effects of domestic drugs may be greater. The benefit of taking brand drugs came with less side effects.” (P4).

“From my persprective, people still felt that branded drugs are better in all ways and comes with less side effects.” (P16).

There were concern among two participants that switching to a NVBP drugs might have an impact on the disease process and cause it to be worsen.

“I feel like the efficacy of domestic drugs is not as good as branded drugs. After taking the domestic drug, his main blood vessel might be blocked again due to the poor efficacy of the drug, which would be serious.” (P1).

The quality of medicines is also a major concern for patients(five participants) who thought that the quality of domestic medicines might not be as good as branded medicines.

“We don't know if the quality of domestically produced medicines is good or not. The biggest doubt is the quality” (P4).

“I think that the quality of branded drugs may be better because of more stringent requirements for various standards.” (P16).

#### Subtheme 1.3- Economy

80.9% patients (17/21) felt that the NVBP drugs were much cheaper than their previous medication.

“It costs about a thousand Yuan per month to take branded drugs, while the cost of domestic drugs would be reduced by about 300 per month” (P1).

“Previously, I was spending over 1000 yuan every three months(season), now I've cut several hundred.” (P7).

In addition, one patient with serious medical conditions felt an increase in drug expenses because the NVBP drugs were not included in the reimbursement list。

“This drug is currently not in the reimbursement list, that is to say, I usually didn't need to pay before, but now I need to pay CNY 33 yuan.” (P8).

A patient with serious medical conditions is also worried about that taking domestic drugs would worsen their symptoms, resulting in the increase of treatment expense in that scenario.

“On the other hand, if my illness gets worsen and I need to be hospitalized again due to taking domestic drugs, I would spend more on medication in long run.” (P1).

#### Subtheme 1.4- Suitability

Three respondents believed that the NVBP drugs could meet demands for some common diseases in the early stages.

“In fact, the NVBP drugs is well qualified, so there are few problems related to this basic disease.” (P8).

However, there is one patient with serious medical conditions who have shown concern about NVBP drugs.

“I want to take the best medicine (the branded drug) and keep it stable all the time, I don't want to change to use domestic medicine.” (P1).

“I wouldn't like to take domestic drugs if I was seriously ill. After all, domestic drugs are not as good as brand ones” (P21).

#### Subtheme 1.5- Accessibility

All participants (*n* = 21) obtained their NVBP drugs in hospital and chose to purchase them again in hospital.

“The first time I bought this medicine was at the hospital, and it is convenient to get the medicine since I have to come back the hospital for the checkups all the time.” (P20).

“I've asked at the community pharmacists before and they don't have this domestic medicine, so I come to the hospital for a prescription every time.” (P21).

### Theme 2: Patient's family and social conditions

#### Subtheme 2.1-Family background

Eight participants interviewed were resistant to switch to NVBP drugs because of their family's financial abundance and strong support from their families.

“I have the ability to earn money and I am willing to spend it to choose better medicines.” (P1).

#### Subtheme 2.2- Surrounding conditions

Two participants were influenced by their surrounding conditions and had a hint of concern about switching to NVBP drugs.

“When I checked online, netizens said that domestic drugs are worse than branded ones” (P16).

#### Subtheme 2.3- Advice from medical staffs

Due to the implementation of the NVBP policy, doctors and pharmacists usually recommend patients to switch to NVBP drugs. 52.3% Patients (11/21) generally obey their doctors and pharmacists due to the trust, which is the main reason why they change their medications.

“I only follow my doctor's advice. He told me there were a shortage of original medicines and recommended to use this domestic medicine as a substitute.” (P3).

“I collected the medicine and found it was different from the previous one. The pharmacist then told me that the ingredients and efficacy were the same as the previous medicine, so I was relieved.” (P20).

#### Subtheme 2.4- Patients' Perception of NVBP Policy

When asked about the NVBP policy, respondents said they had not heard of it or were not familiar with it. Seven participants believe that the NVBP policy has led to price reductions for medicines and reduced the financial burden of patients. In addition, five respondents said that they should reserve the right to choose between NVBP drugs and branded drugs although the implementation of the NVBP policy.

“I think it's progressing too fast, or allowing patients to make their own choices. Or reimburse a smaller percentage, which I can afford.” (P1).

“We as diabetics suddenly have to change to use domestic drugs and the medication regimen I have just adjusted has to be changed again.” (P6).

#### Subtheme 2.5- Reimbursement Policy

Because of the large reimbursement ratio, four patients are not sensitive to the reduced drug expenses by switching to NVBP drugs. Therefore, they are also not as receptive to switching to NVBP drugs.

“I think I would keep taking brand medicine. Because we only need to pay 40% by ourselves with a 60% reimbursement” (P1).

“Because our organization is able to reimburse a large proportion for the chronic illness expenses, we did not feel a noticeable reduction of medical costs” (P14).

As seventeen patients pay by themselves, they can feel the reduction in drug expenses brought about by the NVBP drugs and readily accept the change.

“I don't get reimbursed and pay for my own medication. A medicine used to be over CNY 37 yuan, but now it's CNY 9.80 yuan. That's a much better deal.” (P19).

### Theme 3: medication habits of patients.

#### Subtheme 3.1- brand dependence

Eight respondents have developed a stronger reliance on branded drugs, thus reducing their acceptance of switching to NVBP drugs. And they believe that the advanced technology and higher prices of foreign companies make the quality of medicines better than domestic ones.

“To be honest, I'd rather eat branded ones than domestic ones.” (P15).

“When comparing domestic and branded drugs, everyone thinks that the domestic ones are worse than the branded ones. This has been the general consensus for many years.” (P16).

#### Subtheme 3.2- Inertia in medication

Five patients get used to their original medication that would always prefer the original branded drugs. The stability of the disease makes them stay in their 'comfort zone' instead of switching to NVBP drugs.

“I've been taking the branded medicine for ten years and my blood pressure has been stable. So, I really don't want to change to use another medication” (P2).

“Our factory has a kidney transplant patient whose disease was relatively well controlled by taking branded drugs. But when he switched to domestic drugs, his kidneys didn't work. I really think this policy is a bit unfriendly.” (P6).

#### Subtheme 3.3- Medication confusion

After switching to NVBP drugs, the dosage form and specifications of the medicines that patients are taking may be different which cause confusion for the patients (*n* = 4), In the absence of professional guidance, patients may experience confusion in taking their medication and poor compliance.

“On one occasion, the doctor prescribed two different brands of nifedipine tablets. Is that delayed-release tablet to be taken in the same way as the brand medicine I was taking before?” (P1).

## Discussion

Since the implementation of the NVBP policy, an increasing number of multi-level studies were conducted in recent years, mainly including: study of the rules for the NVBP drugs [31, 32], assessment of the efficacy of the implementation of the NVBP drugs [33] and study on the impact of NVBP drugs on the healthcare industry [34]. Recently, many scholars have used pharmacoeconomic research methods to study the impact of the NVBP drugs on health expenditure [30, 35, 36]. However, few studies have been reported about patients' perceptions of the NVBP policy and attitude towards switching to NVBP drugs and the possible reasons behind it. Through in-depth interviews with patients in this study, we found that: 1) 71.4% patients (15/21) showed a positive attitude towards switching to NVBP medicines; 2) 80.9%patients (17/21) have felt a significant reduction in their medication cost after the implementation of the NVBP policy; 3) Advices from healthcare professionals and health insurance reimbursement policies showed great impacts on patient acceptance of switching to NVBP drugs; 4) Attitudes towards switching to the NVBP drugs varied considerably among patients with different severities of disease.

### Reasons for the patient's positive attitude

The difference in cost between original drugs and NVBP drugs is the main reason for patients' positive attitude towards NVBP drugs. This is similar to the reasons why patients accept generic substitution abroad, which 41% of patients told they would not replace the original drug with a generic if there was no significant price difference [37]. 80.9% patients (17/21) have felt the significant reduction in drug prices. Nevertheless, during the interviews, it was found that one patient felt that medical costs were increased because some NVBP drugs were not be reimbursed by insurance. In addition, advices from health staffs can show great impacts on patients to accept NVBP drugs. If patients do not consult with their doctors, they will complain, "Why is it different from the previous medicine" after the switch to the NVBP drugs. After a brief explanation of the ingredients, quality, efficacy and price of the medicine, many patients readily accept it because they trust the information provided by the pharmacist. This finding is similar to the foreign studies, which enhancing professionals' knowledge of generic drugs could have a positive impact on consumers [38]. However, only 67.3% of pharmacists believe that drugs that have passed the consistency evaluation would have the same efficacy and safety as the original drug [20]. Therefore, there is a need to provide more education on NVBP policies for health practitioners to enhance their feeling of recognition. Secondly, there is also a need to improve the confidence of health practitioners by carrying out scientific education about the consistency evaluation of NVBP drugs.

### Reasons for the patient's negative attitude

The main reasons for patients' negative attitudes towards switching to NVBP drugs are concerns about the safety and efficacy of medicines and changes in patients' medication habits. While some of the original branded drugs have also been selected in the list of NVBP drugs, the majority of the NVBP drugs are generics. In China, patients generally refer to NVBP drugs as "domestic" or "generic", and most patients believe that domestic drugs are not as good as the original drugs in terms of quality and efficacy because of their low price. In addition, a few patients who have experienced the side effects of domestic drugs or have experienced poor treatment with domestic drugs have expressed doubts about the safety and efficacy of all NVBP drugs. Other academics have also found in generic substitution studies that about a third of patients told they experience uneasiness and additional side effects [39]. Furthermore, some medical institutions have curtailed the supply of original branded drugs in order to reduce patients' drug expenses and to complete the task volume of NVBP drugs [40]. Patients are reluctant to switch to NVBP drugs due to their trust in original branded medicines, and avoiding the potential confusion after switching to NVBP drugs. Therefore, there is an urgent need for medical practitioners and key media to promote the quality, efficacy and price of NVBP drugs to patients, thereby improving their perceptions and boosting their confidence in NVBP drugs. Moreover, while implementing the NVBP policy, medical practitioners should also strengthen the communication with patients, respect their perferences and use a more scientific approach to guide them to accept NVBP medicines.

### Diseases with different severities is one of the main influencing factors

In this study, we found that patients' attitudes towards switching to NVBP drugs vary considerably among patients with different severities of disease. Firstly, 28.6% patients (6/21) with only one chronic disease, (e.g. Grade 1 hypertension) showed positive attitudes towards switching to the NVBP drugs. Because they feel that these drugs are well qualified and that taking them over a long period of time can significantly reduce the drug costs. Secondly, 28.6% patients (6/21)who taking a combination of 2–3 underlying diseases but without complications expressed caution about switching to NVBP drugs. On the one hand, they are concerned that the less-than-expected therapeutic effect of the NVBP drugs may lead to complications; on the other hand, switching to the NVBP drugs can drastically reduce drug expenses. Finally, 23.8% patients (5/21) who have experienced bleeding or ischemic critical illnesses have developed a rejected attitude towards switching to NVBP drugs. The main reasons for this may be: 1) patients are concerned that the poor efficacy of NVBP drugs may result in their death or worsening of existing symptoms; 2) because of the large percentage reimbursement for serious illnesses, the switch to NVBP drugs is not a significant reduction in their expenditure; 3) the absence of NVBP drugs from the reimbursement list may lead to increased cash outlay for patients. Therefore, real-world evaluation studies of NVBP drugs are essential, especially for diseases with different severities that require stratified assessment so that the NVBP drugs can be implemented with greater precision.

Economy, efficacy and safety of medicines are the main concerns of patients. The NVBP policy has initially solved the problem of inflated drug prices, but there is still a need to further expand the scope of NVBP drugs so that the policy benefits more patients.

### Strengths and limitations

To our knowledge, this is the first study in China that investigated patients' attitudes and opinions towards NVBP drugs. This study also revealed factors that influence patients' acceptance of switching to NVBP drugs. This study also identified several areas for improvement in the further implementation of the NVBP policy. The findings of the study have profound implications for the implementation of NVBP policies in China. Therefore, this study fills a gap in the current researchers on NVBP drugs. This study has several limitations. Due to the nature of the qualitative design, the results of the study may not be generalized to all patients. This study only included participants from one hospital. Even if The hospital is a large general hospital covering all departments, it still cannot represent views of all the patients. Participants are expressed in Chinese. Although we have tried our best to improve the validity and minimize the risk of losing meaning, there was still the possibility of ambiguity in language conversion.

## Conclusion

The implementation of the NVBP policy has significantly reduced the expenditure of patients and has been supported by the majority of patients. However, during the implementation of the NVBP policy, this study has also identified a number of issues that need to be improved, such as: 1) improving patients' perceptions and confidence in NVBP drugs by educating patients through health practitioners and key media, 2) medical practitioners should also strengthen communication with patients, respect their preference and use a more scientific approach to guide them to accept NVBP drugs, 3) real-world evaluation studies of NVBP drugs are essential, especially for diseases with different severities that require stratified clinical assessment.

## Data Availability

The dataset generated during and analysed during the current study are available from the corresponding author on reasonable request.

## Abbreviations

NVBP: National Volume-Based Procurement
GPOs: Group purchasing organizations
GDP: Gross Domestic Product
CNY: Chinese yuan
COREQ: Consolidated Criteria for Reporting Qualitative Studies

## References

[1] Hassali MA (2014). The experiences of implementing generic medicine policy in eight countries: A review and recommendations for a successful promotion of generic medicine use. Saudi Pharm J.

[2] Kesselheim AS, Avorn J, Sarpatwari A (2016). The high cost of prescription drugs in the united states: origins and prospects for reform. JAMA.

[3] Wouters OJ, Kanavos PG (2017). A comparison of generic drug prices in seven European countries: a methodological analysis. BMC Health Serv Res.

[4] Wouters OJ, Kanavos PG, McKEE M (2017). Comparing Generic Drug Markets in Europe and the United States: Prices, Volumes, and Spending. Milbank Q.

[5] Parkinson B (2015). Disinvestment and Value-Based Purchasing Strategies for Pharmaceuticals: An International Review. Pharmacoeconomics.

[6] Smith C (2004). Retail Prescription Drug Spending In The National Heaith Accounts. Health Aff (Millwood).

[7] Hu Q, Schwarz LB, Uhan NA (2012). The Impact of Group Purchasing Organizations on Healthcare-Product Supply Chains. Manuf Serv.

[8] Brock TH (2003). Hospitals, group purchasing organizations, and the antitrust laws. Healthc Financ Manage.

[9] Shao R, Xie J, Jiang R (2014). Analysis on the group purchasing organizations in the United States and its implications for Chinese drug procurement. Chin J Health Policy..

[10] Kun Z, Shangxi L, Liangchu Y (2022). Analysis of China's Health Fiscal Expenditure in the New Century. FISCAL SCIENCE.

[11] Liu xiaojing, Talking about the significance of traditional Chinese medicine services in public hospitals to reduce medical expenses for patients. (Published in Chinese) Contemporary Medical Symposium, 2015;13(21):5–6.

[12] 2020 Health Statistics Yearbook. 2021. Available: http://www.nhc.gov.cn/mohwsbwstjxxzx/tjtjnj/202112/dcd39654d66c4e6abf4d7b1389becd01.shtml [Accessed 21 Dec 2022]

[13] Notice of the General Office of the State Council on printing and issuing national organizational drugs for centralized procurement and use of pilot solutions. (Published in Chinese) Gazette of the State Council of the People's Republic of China, 2019(03): p.12–15. Available: http://www.gov.cn/gongbao/content/2019/content_5361793.htm [Accessed 20 Dec 2022]

[14] Hu Shanlian, How to procure the amount of medicine towards normalization and institutionalization. (Published in Chinese) Medicine Economic News, 2021.p.2. 10.38275/n.cnki.nyyjj.2021.000232)

[15] China Food and Drug Administration. Evaluation scheme of generic quality consistency. China food and drug administration, 2013. Available: https://www.nmpa.gov.cn/xxgk/fgwj/gzwj/gzwjyp/20130216144301191.html [Accessed 30 June 2022].

[16] Yuan J (2021). Lowering drug prices and enhancing pharmaceutical affordability: an analysis of the national volume-based procurement (NVBP) effect in China. BMJ Glob Health.

[17] Yang Y (2021). The impact of "4 + 7" volume-based drug procurement on the volume, expenditures, and daily costs of antihypertensive drugs in Shenzhen, China: an interrupted time series analysis. BMC Health Serv Res.

[18] He Jiangjaing, et al., The impact of national organizational drug centralized procurement and use on the management and use of clinical medication. (Published in Chinese) Chin Health Resources, 2021. 24(01):29–31. 10.13688/j.cnki.chr.2021.200802)

[19] Du Z (2022). Reevaluation of adverse drug reactions of psychiatric drugs under the chinese drug volume-based procurement policy. BMC Health Serv Res.

[20] Qu J (2021). Knowledge, perceptions and practices of pharmacists regarding generic substitution in China: a cross-sectional study. BMJ Open.

[21] Jinping XIE (2021). Analysis of Key Stakeholders in the National Drug Centralized Procurement Policy. China Pharmacy.

[22] Bradshaw C, Atkinson S, Doody O (2017). Employing a qualitative description approach in health care research. Glob Qual Nurs Res.

[23] Considine J (2020). Understanding the patient experience of early unplanned hospital readmission following acute care discharge: a qualitative descriptive study. BMJ Open.

[24] Tong A, Sainsbury P, Craig J (2007). Consolidated criteria for reporting qualitative research (COREQ): a 32-item checklist for interviews and focus groups. Int J Qual Health Care.

[25] World Medical Association Declaration of Helsinki. Ethical principles for medical research involving human subjects. Eur J Emerg Med. 2001;8(3):221–23. 10.1097/00063110-200109000-00010 11587468

[26] Hassali MA, Kong D, Stewart K (2006). Generic medicines: Perceptions of general practitioners in Melbourne, Australia[J]. J Generic Med.

[27] Sharrad AK, Hassali MA (2011). Consumer perception on generic medicines in Basrah, Iraq: Preliminary findings from a qualitative study[J]. Res Social Adm Pharm.

[28] Braun V, Clarke V (2006). Using thematic analysis in psychology. Qual Res Psychol.

[29] Edward KL, Welch T (2011). The extension of Colaizzi's method of phenomenological enquiry. Contemp Nurse.

[30] Northall T (2020). The application and tailoring of Colaizzi's phenomenological approach in a hospital setting. Nurse Res.

[31] JIANG Chang-song, et al., Analysis on Some Economic Principles of Volume-based Procurement. Health Economics Research, 2021;38(06):63–65.

[32] Li Yiren, Lu Yun, Chang Feng, Research on Rules of Drug Quantity Purchase Based on Enterprise Quotation Game. Price:Theory & Practice, 2020;(01):74–77+177.

[33] Li Shouxi, Shen Tingzhi, Study on the Effect of Implementing the Policy of Volume-based Procurement in China——Analysis of the impact on the performance and innovation ability of pharmaceutical enterprises. Price:Theory & Practice, 2020;(11): 49–52+97.

[34] Yushu H, Libo T (2020). The Influence of Drug Centralized Group Procurement Policy to Chinese Pharmaceutical Industry——An Analysis Based on Perspective of Industrial Economics. China Health Insurance.

[35] Wang N (2021). Influence of Chinese National Centralized Drug Procurement on the price of policy-related drugs: an interrupted time series analysis. BMC Public Health.

[36] Yuan J (2021). Lowering drug prices and enhancing pharmaceutical affordability: an analysis of the national volume-based procurement (NVBP) effect in China. BMJ Glob Health.

[37] Kjoenniksen I, Lindbaek M, Granas AG (2006). Patients' attitudes towards and experiences of generic drug substitution in Norway. Pharm World Sci.

[38] Hakonsen H (2009). Generic substitution: additional challenge for adherence in hypertensive patients?. Curr Med Res Opin.

[39] Hkonsen H, T.E.L.A. A review of patient perspectives on generics substitution: what are the challenges for optimal drug use[J]. Generics Biosimilars Initiative J. 2012;1(1):28–32.

[40] Menglijun, Analysis on the Pilot Operation of Drug Centralized Procurement and Use in Shenyang. China Health Insurance, 2019(08):28–31.

